# Impact of placental *Plasmodium falciparum* malaria infection on the Cameroonian maternal and neonate’s plasma levels of some cytokines known to regulate T cells differentiation and function

**DOI:** 10.1186/s12936-016-1611-0

**Published:** 2016-11-21

**Authors:** Jean Claude Djontu, Stalone Siewe Siewe, Yolande Delphine Mpeke Edene, Benderli Christine Nana, Edwige Vanessa Chomga Foko, Jude Daiga Bigoga, Rose F. G. Leke, Rosette Megnekou

**Affiliations:** 1 Department of Animal Biology and Physiology of the Faculty of Sciences, University of Yaoundé I, P.O. Box 812, Yaoundé, Cameroon; 2 The Biotechnology Center, University of Yaounde I, P.O. Box 3851, Messa, Yaoundé, Cameroon; 3 Department of Biochemistry of the Faculty of Sciences, University of Yaoundé I, P.O. Box 812, Yaoundé, Cameroon

**Keywords:** *Plasmodium falciparum*, Placental malaria, Cytokines, Pregnant women, Birth weight, Cameroon

## Abstract

**Background:**

The impact of placental malaria (PM) infection on the expression profile of some cytokines known to regulate T cell differentiation and function and their influence on birth weight remain unclear. Moreover, there are no reports showing the relationship between PM and IL-27 or IL-28A. This study therefore sought to investigate whether placental *P. falciparum* infection alters the expression profile of the cytokines IL-28A, IL-27, IL-17E and IL-6 in mothers and their new born.

**Methods:**

In a cross-sectional study conducted between 2013 and 2015 in Yaoundé, Cameroon, peripheral, placental and cord blood samples were collected from 108 women at delivery. Parasitaemia was determined microscopically and haemoglobin levels determined using a Coulter counter. Plasma levels of cytokines (IL-28A, IL-27, IL-17E and IL-6) were measured by Luminex magnetic screening assay.

**Results:**

Malaria parasite density in placenta impression smear associated negatively with maternal haemoglobin level (P < 0.0001) and baby birth weight (P = 0.016). While IL-17E, IL-27 and IL-28A levels were significantly higher in placental and cord plasma than in peripheral (P < 0.0001, < 0.001 and P = 0.026, respectively), an opposite relationship was observed with IL-6 (P = 0.0018). Multivariate analysis confirmed results of univariate analysis where the presence of malaria parasites or pigments in placenta tissue impression smears correlated with decrease levels of maternal IL-17E, IL-27 and IL-28A and neonate levels of IL-28A and IL-17E (0.0001 ≤ P ≤ 0.02). Placental and peripheral parasitaemias also correlated positively with peripheral plasma levels of IL-6 (r_s_ = 0.18, P = 0.05 and r_s_ = 0.17, P = 0.07, respectively). In addition, high maternal haemoglobin level associated with increasing levels of IL-17E, IL-27 and IL-28A in peripheral plasma (0.002 ≤ P ≤ 0.018) and high placental and cord plasma levels of these cytokines associated with increasing birth weight (0.0001 ≤ P ≤ 0.0027).

**Conclusions:**

Placental malaria downregulates maternal plasma levels of IL-17E, IL-27 and IL-28A and neonates’ plasma levels of IL-17E and IL-28A cytokines, which could help for parasite clearance and increase child birth weight. The study is expected to provide leads that should help identify potential biomarkers for improved birth weight and therapeutic interventions.

## Background

During pregnancy, *Plasmodium*-infected erythrocytes (IEs) accumulate in the placenta [1, 2], inducing attraction of some leucocytes in the organ, pathological changes that alter the materno-fetal exchange system and result into intra-uterine growth retardation and low birth weight [3]. It also alters cytokine expression profile [4–7]. Although cytokines play a crucial role in pathogen clearance, they could also elicit adverse outcomes in several infectious diseases if their secretion is not well regulated. Thus, accumulation of IEs in the placenta can alter the anti-inflammatory/pro-inflammatory cytokines balance, which might play a dominant role in the pathophysiology of placental malaria (PM) [5, 8]. Indeed, a successful pregnancy is characterized by the predominance of anti-inflammatory cytokines at the feto-maternal interface that downregulates inflammatory response, which could be detrimental to the fetus [9]. Also, the accumulation of *Plasmodium falciparum*-infected erythrocytes and leucocytes in the placenta is associated with alteration in cytokine expression and some oxidative stress biomarkers profiles [10, 11]. The infiltrated leukocytes are the principal sources of inflammatory cytokines production [8, 12]. Among the cytokines produced, IL-27 and IL-6 can elicit both pro-inflammatory and anti-inflammatory effects. In fact, IL-27 has been described to induce T helper 1 (Th1) cell associated transcription factor T-bet, which enhances Th1 differentiation [13, 14], while other studies showed that it inhibits the development of IL-17-producing helper T (Th-17) cells [15] and decreases Th2 response [16]. IL-6 cytokine inhibits transforming growth factor-β (TGF-β)-induced IL-10-producing regulatory T cell (Treg) differentiation [17] but together with (TGF)-β, it preferentially promotes the differentiation of IL-17-producing T helper cells (Th17) [18]. IL-17E (IL-25) can be produced by mucosal epithelial cells as well as many immune cell types, including mast cells, macrophages, eosinophils, Th2 cells, and NKT cells [19, 20]. It is involved in Th2 cell-promoting cytokine [21] and suppresses Th17 cell responses. As regards IL-28A, previous studies presented this cytokine as a newly designated type III interferon (IFN)-λ, having antiviral effects [22, 23] and produced principally by antigen-presenting cells (APCs) [24]. This cytokine can improve the Th17-induced inflammation, provide Th1 polarization and antagonize Th2 responses [25]. Therefore, IL-6, IL-27, IL-17E, and IL-28A are critical cytokines possibly involved in the regulation of human immunological diseases induced by Th1, Th2, Treg, and Th-17 cells response imbalance [26].

As observed previously during PM, CXCL-10 chemokine might attract monocytes and lymphocytes into the placenta where they produce cytokines (IL-10 and IL-17A) whose levels affect parasite clearance and modulate the disease [10]. In addition, high plasma levels of some inflammatory cytokines, such as Th1 cells-IFN-γ and Th-17 cells-IL-17A, have been shown to correlate with the absence of pregnancy-associated malaria in Cameroonian women [27], while over-expression of IL-10 may be associated with the persistence of malaria parasites in the placenta leading to low birth weight babies [8, 10]. In addition, some studies have shown that IL-27-producing CD4+ T cells may play a role in the regulation of protective immune response against malaria [28]. Although the differentiation and function of IFN-γ, IL-10 and IL-17A-producing T cells are critically influenced by IL-6, IL-27, IL-17E, or IL-28A cytokines, the impact of placental *P. falciparum* infection on the expression profile of these regulatory cytokines in women at delivery and its influence on birth weight is unknown. Therefore, this study was carried out to better understand the alteration in cytokine homeostasis in women with PM and its consequences on birth weight. The study is expected to provide leads that should help identify potential biomarkers for improved birth weight and therapeutic interventions.

## Methods

### Study site and population

Between May 2013 and January 2015, a cross-sectional survey was carried out at the Marie Reine Health Centre in Etoudi, Yaoundé, Cameroon, to evaluate the impact of placental *P. falciparum* infection on the expression profile of some regulatory cytokines in women at delivery and its influence on birth weight. The study site and population as well as the sample collection have been described elsewhere [10]. Peripheral, placental and cord blood samples were aseptically collected in EDTA tubes from 108 women aged 16–39 years immediately following delivery. A portion of each sample was used to prepare smears for malaria microscopy. The remainder was centrifuged and the plasma stored at −80 °C for cytokine measurements. Placental tissues were also collected and used to prepare impression smears and for histology. The study participant characteristics are summarized in Table 1.

**Table 1:** Characteristics of the study population

Parameters	All women (n = 108)	PM + women (n = 24)	PM-women (n = 84)	P value
Age, median (range), years	25 (16–37)	25 (16–37)	26 (17–37)	0.2
Primipara	39 (36%)	9 (23%)	30 (77%)	
Secondipara	26 (24%)	8 (30%)	18 (70%)	
Multipara	43 (40%)	7 (16%)	36 (84%)	
Hb levels (g/dL)	12.6 (7.5–15.7)	10.7 (7.5–12)	12.9 (10–15.7)	<*0.0001*
Baby birth weight (g)	3350 (1900–4850)	3200 (2100–3700)	3400 (1900–4850)	*0.016*
Malaria by RDT	28 (26%)	23 (82%)	5 (18%)	
Malaria by peripheral blood smears	19 (18%)	19 (100%)	0 (0%)	
Malaria by placental blood smears	15 (14%)	15 (100%)	0 (0%)	
Placental malaria by histology	16 (15%)	16 (100%)	0 (0%)	
Malaria pigments	14 (13%)	13 (93%)	1 (7%)	

Mann–Whitney Rank Sum Test was used to compare different variables between infected and non-infected women

Values in the parentheses represent either percentage or range

Significant p values are in italics (p ≤ 0.05)

*PM* placental malaria negative women; *PM* + placental malaria positive women

### Diagnosis of placental malaria and determination of haemoglobin levels

For malaria microscopy, maternal peripheral, placental and cord blood were used to prepare thick and thin blood smears while placenta tissue was used to prepare impression smears. Slides were stained using Giemsa and examined for the presence of malaria parasites. *Plasmodium falciparum* infection was further confirmed using the rapid diagnostic test, malaria Carestart™ HRP2 (Pf) (Access Bio Inc, NJ, USA). Placental tissue sections were fixed in buffered formalin, embedded, stained with haematoxylin-eosin, and examined for the presence of parasites and malaria pigments. A woman was considered placental *P. falciparum* positive if infected erythrocytes were detected in impression smears of placenta tissue, placental blood smear or in histological sections. The presence of malaria pigment in placental tissue was declared when detecting pigment within monocytes from placental impression smear or/and in intervillous spaces of placental histological sections. Haemoglobin (Hb) levels in maternal blood and neonate cord blood were determined using a Coulter counter (URIT-3300, Europe). A woman was considered anaemic if Hb < 11 g/dL.

### Measurement of cytokines

Plasma levels of IL-6, IL-17E, IL-27, and IL-28A were measured by magnetic luminex screening assay, using Human Premixed Multi-Analyte Kit (R&D Systems, Inc. Minneapolis, MN, USA). Human, magnetic, premixed, microparticle cocktail of antibodies specific to IL-6, IL-17E, IL-27, and IL-28A was used to simultaneously screen maternal peripheral, placental and neonate cord plasma. The assay was carried out according to manufacturer’s instructions. Briefly, plates were incubated on the horizontal shaker with 50 µL/well of different diluted samples and concentration of standard cytokines provided alongside with 50 µL of human, magnetic, premixed, microparticle cocktail with antibodies specific for each cytokine. After washing using magnetic plate separator (Luminex, Austin, TX, USA, Cat# CN-0269-01), plates were incubated with 50 µL/well of human premixed biotin-antibodies cocktail specific for each cytokine. Antibody-cytokine complexes were revealed using Streptavidin-PE. Plates were read using a Luminex MAGPix Analyzer (XMAP Technology, SN, USA) and results were expressed as median fluorescence intensity (MFI). A standard curve was generated for each cytokine to convert MFI into corresponding cytokine relative concentration. The sensitivity of the assay for each cytokine was: 0.2 pg/ml (IL-6), 4.5 pg/ml (IL-17E), 3.6 pg/ml (IL-27), and 8.4 pg/ml (IL-28A), which was the minimum detectable concentration of each cytokine.

### Statistical analyses

The GraphPad Prism 5.03 software was used for statistical analyses. Results were reported as medians with 95% confidence intervals. Mann–Whitney rank sum test was used to evaluate inter-group differences in the levels of cytokines. Spearman rank order coefficient (r_s_) was used to evaluate parameter association. Regression analysis was used to identify significant association between cytokines tested and other parameters. Proportions were compared using Fisher’s exact test. *P* values <0.05 were considered statistically significant.

## Results

### Study population

The baseline characteristics of the study participants are summarized in Table 1. Twenty-four (22.5%) of the women were positive for placental *P. falciparum* infection amongst whom 15 (62.5%) were positive for the placental and peripheral blood as well as the impression smears of placental tissue; four (16.66%) were positive for the peripheral blood and impression smears of placental tissue, while five (20.8%) were positive only for the impression smears of placental tissue. With regards to malaria rapid diagnostic test (RDT), 28 out of the 108 women (26%) were positive, of which 23 (82%) were women with infected placenta. These observations confirm that multiparous women (16%) were less likely to have placental malaria compared to the secondiparous (30%) (P = 0.02) and primiparous (23%) (P = 0.2). There was no significant difference in median age between *P. falciparum*-infected and non-infected women (P = 0.2). Hb level was lower in malaria-infected women compared to non-infected (P < 0.0001). Birth weight from women with infected placenta was lower compared to the non-infected (P = 0.016). Histological studies showed that malaria pigment in placental tissue was more frequent among infected women compared to non-infected women (P < 0.0001). In fact, malaria pigment in placental tissue was found in 13 (54.1%) of placental malaria-positive women, while only one woman (1.1%) was found to have malaria pigment among the placental malaria negative women.

Parasitaemia was observed to increase significantly with decreasing peripheral and cord blood Hb levels as well as baby birth weight [(r _s_ = −0.55, −0.23 and −0.25), P < 0.0001, 0.015 and 0.011, respectively)]. A similar but non-significant trend was observed with parity and mother’s age [(r_s_ = −0.10 and −0.14), P = 0.32 and 0.16, respectively)]. These results confirm that babies born from infected mothers are more likely to have low birth weight compared to those non-infected.

### Plasma level of IL-17E, IL-27 and IL-28A cytokines are elevated in the placental and cord blood while IL-6 is higher in peripheral blood

**Fig. 1:**
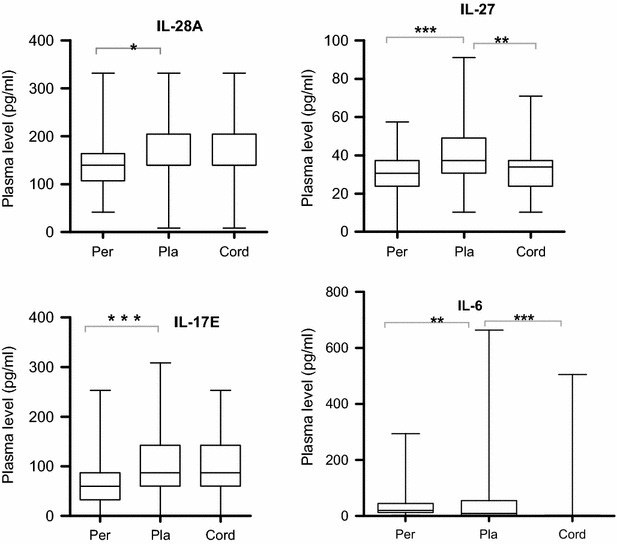
Changes in plasma levels of cytokines between peripheral, placental and cord blood at delivery. Plasma levels of IL-28A, IL-27, IL-17E, and IL-6 cytokines in peripheral blood were compared with that in placental and cord blood from Cameroonian women using Mann–Whitney Ran Sum test. *Box* represents median with 25 and 75 percentiles. *Per* peripheral blood; *Pla* placental blood; Cord blood (N = 108). *P < 0.05; **P ≤ 0.001; ***P ≤ 0.0001

In order to determine whether the profile of the cytokines varied with the collection sites, levels of IL-6, IL-17E, IL-27, and IL-28A in the peripheral plasma were compared with those in placental and cord plasma. The plasma levels of IL-17E, IL-27 and IL-28A were significantly higher in placental than in peripheral plasma (P < 0.0001, < 0.001 and P = 0.026, respectively), while levels of IL-6 were instead higher in the peripheral plasma (P = 0.0018) (Fig. 1; Table 2). Although there was no significant difference in the levels of IL-17E and IL-28A between placental and cord plasma, IL-27 and IL-6 levels were significantly higher in placental plasma than in cord plasma (P = 0.0010 and P < 0.001, respectively). These results suggest that IL-17E, IL-27 and IL-28A are synthesized in the placenta with IL-28A and IL-17E crossing the placenta barrier, leading to their observed high levels in cord blood.

**Table 2:** Cytokine levels between peripheral, placental and cord plasma

Cytokines (pg/mL)	Peripheral plasma	Placental plasma	Cord plasma
IL-28	1395 [1069–1637]	1395 [1395–2040]	1395 [1395–2040]
IL-27	3057 [2384–3729]	3729 [3057–4905]	3393 [2384–3729]
IL-17 E	5971 [3209–8733]	8733 [5971–14260]	8733 [5971–14260]
IL-6	1984 [1169–4495]	981 [328–5498]	127 [026–277]

Values outside the bracket correspond to the median while values in bracket correspond to inter-quartile range

### *Plasmodium falciparum* parasiteamia correlated negatively with maternal levels of IL-17E, IL-27, and IL-28A, but positively with IL-6

**Fig. 2:**
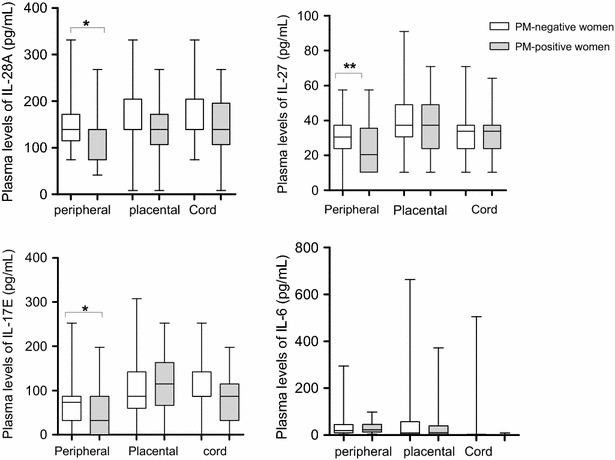
Plasma levels of IL-28A, IL-27, IL-17E and IL-6 cytokines in PM-negative and PM-positive women. Plasma levels of IL-28A, IL-27, IL-17E, and IL-6 cytokines were compared between PM-negative (N = 84) and PM-positive (N = 24) women using Mann–Whitney Rank Sum test. *Box* represents median with 25 and 75 percentiles. * P < 0.05; **P ≤ 0.001. *PM* Placental malaria

To investigate whether placental malaria influences the expression profile of IL-28A, IL-27, IL-17E and IL-6 in maternal and neonate blood, the levels of these cytokines were compared between women with infected placenta and those without. Women with infected placenta had lower levels of IL-28A, IL-27 and IL-17E in peripheral plasma compared to their non-infected counterparts (P = 0.03, 0.005 and 0.03, respectively (Fig. 2). In addition, the levels of these cytokines in peripheral plasma correlated negatively with the parasitaemia from placenta tissue impression smear [(r_s_ = −0.20; P = 0.02; r _s_ = −0.28; P = 0.002; r_s_ = −0.21; P = 0.028)], peripheral blood smear [(r_s_ = −0.20; P = 0.03; r_s_ = −0.30; P = 0.001; r_s_ = −0.21; P = 0.029)], respectively, and from the placental blood smear only for IL-27 (r_s_ = −0.23, P = 0.014) (Table 3). Although higher in non-infected women, no significant difference was observed in the cord plasma levels of IL-28A and IL-17E between women with infected and non-infected placenta (Fig. 2). While no difference was observed between the infected and non-infected women for IL-27 and IL-17E placental plasma levels, that of IL-28A was higher in the non-infected women although not significant. In addition, negative correlations were observed between parasitaemias and cord plasma levels of IL-28 A, IL-17E or placental level of IL-28A (Table 3). With regards to IL-6, its peripheral levels correlated positively with placental parasitaemia (r_s_ = 0.18, P = 0.05) and peripheral parasitaemia (r_s_ = 0.17, P = 0.07) (Table 3). Together, these suggest that placental *P. falciparum* infection may be associated with decrease in peripheral plasma levels of IL-28A, IL-27 and IL-17E and increase IL-6 level.

**Table 3:** Correlations between cytokine levels and different parasitaemias

Cytokines	% IRBCs of impression smear (from placenta tissue) (n = 108)		Placental blood parasitaemia		Peripheral blood parasitaemia	
	r_s_	P value	r_s_	P value	r_s_	P value
IL-28 A per	−*0.20*	*0.02*	−0.10	0.28	−*0.20*	*0.03*
IL-28 A pla	−0.15	0.12	−0.12	0.20	−0.12	0.21
IL-28 A cord	−0.15	0.10	−0.14	0.13	−0.11	0.24
IL-27 per	−*0.28*	*0.002*	−*0.23*	*0.015*	−*0.30*	*0.001*
IL-27 pla	−0.05	0.54	−0.003	0.97	−0.03	0.68
IL-27 cord	−0.01	0.83	−0.005	0.95	−0.002	0.98
IL-17 E per	−*0.21*	*0.028*	−0.12	0.19	−*0.20*	*0.029*
IL-17 E pla	−0.002	0.98	−0.05	0.54	−0.02	0.77
IL-17 E cord	−0.14	0.13	−0.14	0.13	−0.10	0.28
IL-6 per	0.09	0.35	*0.18*	*0.05*	0.17	0.07
IL-6 pla	−0.01	0.84	0.04	0.61	0.01	0.85
IL-6 cord	−0.002	0.98	0.07	0.44	0.04	0.64

The Spearman’s rank order correlation test was used

Significant p values are in italics (p ≤ 0.05)

*r*_*s*_ Coefficient of correlation; *IRBCs* infected red blood cells; *Per* Peripheral plasma; *Pla* Placental plasma; *Cord* Cord plasma; *IL* Interleukin

### Chronic placental malaria infection is associated with decrease plasma levels of maternal IL-17E, IL-27, IL-28A and neonate IL-17E and IL-28A

**Fig. 3:**
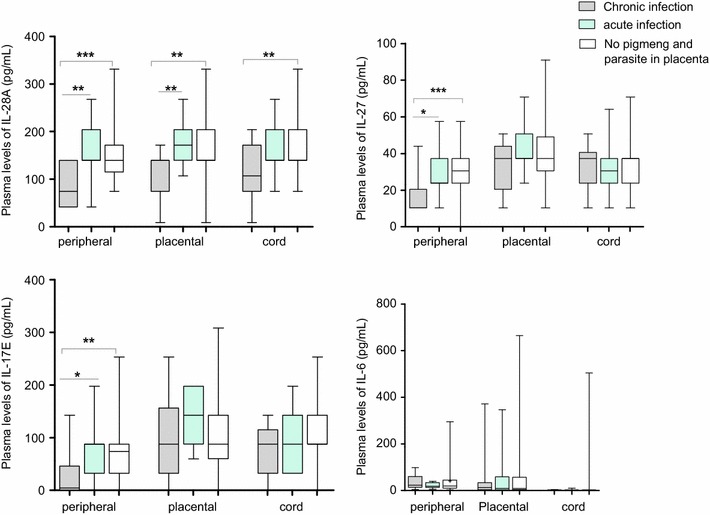
Plasma levels of cytokines in delivering women with chronic infection, acute infection and with no malaria parasite and pigment in the placenta tissue. Plasma levels of IL-28A, IL-27, IL-17E, and IL-6 cytokines were compared between women with chronic infection (N = 13), acute infection (N = 11) and women without malaria parasites and pigment in placenta tissue (N = 83), using Mann–Whitney Rank Sum test. *Box* represents median with 25 and 75 percentile *P < 0.05; **P ≤ 0.001; ***P ≤ 0.0001

According to Rogerson et al. [29], the presence of malaria parasites and pigment in placental tissue of women indicates a chronic infection, while an acute infection is defined as the presence of malaria parasite in placental tissue without pigment. The presence of malaria pigment only indicates past infection. Therefore, the cytokine levels in women with chronic infection (n = 13) were compared to those of women with acute infection (n = 11) and women with no malaria parasite and pigment in placental tissue (n = 83) (Fig. 3). Results showed lower levels of IL-28A in the peripheral, placental and cord plasma of women with chronic infection compared to those with acute infection (P = 0.008, 0.0085 and 0.06, respectively) and those with neither parasite nor pigment in placental tissue (P = 0.0001, 0.0088 and 0.0078, respectively). Levels of IL-17E and IL-27 in peripheral plasma were also lower in women with chronic infection than in those with acute infection (P = 0.058 and 0.021, respectively) and with neither parasite nor pigment in placental tissue (P = 0.0017 and 0.0004, respectively). The level of IL-17E in cord blood was slightly lower in women with chronic infection compared to those with no parasite and pigment in placental tissue (P = 0.057). Regarding IL-6, its level in peripheral, placental and cord plasma was slightly higher in women with chronic infection than in those with infection or with neither malaria parasite nor pigment in the placenta. The difference was also not significant (P > 0.2).

### Maternal haemoglobin level correlated positively with levels of IL-17E, IL-27 and IL-28A in peripheral blood while high levels of these cytokines in placental and cord blood increased with increasing baby birth weight

**Fig. 4:**
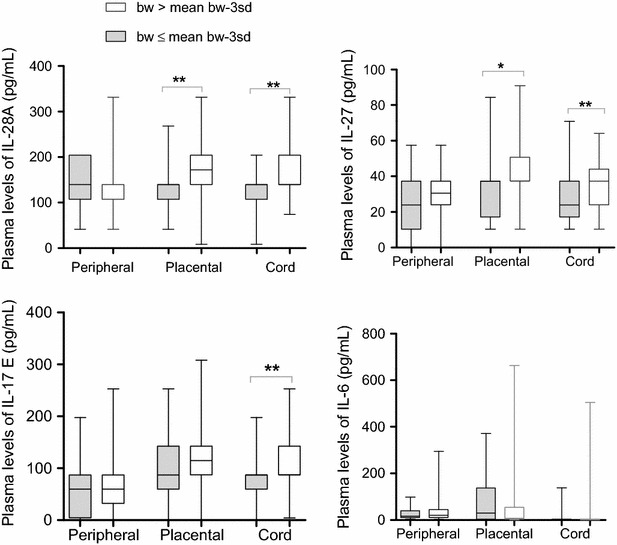
Plasma levels of cytokines in women at delivery in relation with birth weight. Plasma levels of IL-28A, IL-27, IL-17E, and IL-6 cytokines were compared between women having baby with birth weight > mean birth -3Sd (2800 g) (n = 89) and women having baby with birth weight ≤ mean birth -3Sd (n = 19), using Mann–Whitney Rank Sum test. *bw* birth weight. *Box* represents median with 25 and 75 percentile *P < 0.05; **P ≤ 0.005

In this study, multipara women were less likely to be infected by malaria parasites compared to the secondipara and primipara. Also, PM correlated with decrease in maternal Hb level (P < 0.0001) and birth weight (P = 0.016) (Table 1). Therefore, the relationships between levels of assayed cytokines and parameters such as maternal Hb level, baby birth weight, parity, and mother’s age were investigated. Maternal Hb levels correlated positively with the levels of IL-28A, IL-27 and IL-17E in peripheral plasma (0.24 ≤ r_s_ ≤ 0.28; 0.002 ≤ P ≤ 0.012) (Table 4). Placental and cord plasma levels of IL-28A and IL-27 were significantly elevated in women having babies with birth weight higher than mean birth weight of -3SD (2800 g) compared to women having babies with birth weight equal to or lower than mean birth weight of -3SD [(P = 0.0057 and = 0.010, respectively for placental plasma levels of IL-28A and IL-27) and (P = 0.0027 and 0.0053, respectively for cord plasma levels of IL-28A and IL-27)] (Fig. 4). Although the placental plasma level of IL-17E was not significantly different in these two groups of (P = 0.36), its cord plasma level was significantly higher in the group of women having babies with birth weight higher than mean birth weight of-3SD (P = 0.0034). Furthermore, baby birth weight was observed to correlate positively with levels of IL-28A, IL-27 and IL-17E, in placental plasma (r_s_ = 0.29, 0.29 and 0.16; P = 0.002, 0.002 and 0.09), respectively) and cord plasma (r_s_ = 0.40, 0.35 and 0.38; P ≤ 0.0002, for all), respectively (Table 4). Levels of IL-28A and IL-27 in placental plasma (r_s_ = 0.18; P = 0.05) and (r_s_ = 0.23; P = 0.01) as well as cord plasma levels of IL-17E (r_s_ = 0.19; P = 0.04) correlated positively and significantly with parity. Only placental levels of IL-27 associated significantly with mother’s age (r_s_ = 0.20, P = 0.03) (Table 4). None of these parameters associated significantly with levels of IL-6. Together, these results suggest the invaluable roles of IL-28A, IL-27 and IL-17E during pregnancy for normal fetal growth.

**Table 4:** Correlation between cytokine levels and hemoglobin levels, parity, mother’s age and baby weight

Cytokines	Hb per		Birth weight		Parity		Mother age	
	r_s_	P	r_s_	P	r_s_	P	r_s_	P
IL-28 per	*0.24*	*0.012*	−0.01	0.88	0.10	0.28	0.14	0.12
IL-28 pla	0.09	0.34	*0.28*	*0.002*	*0.18*	*0.05*	0.12	0.18
IL-28 cord	0.15	0.10	*0.39*	<*0.0001*	0.07	0.45	0.05	0.56
IL-27 per	*0.28*	*0.002*	0.11	0.23	−0.05	0.60	0.02	0.82
IL-27 pla	0.009	0.92	*0.28*	*0.002*	*0.23*	*0.01*	*0.20*	*0.03*
IL-27 cord	−0.06	0.48	*0.35*	*0.0002*	0.11	0.22	0.03	0.72
IL-17E per	*0.25*	*0.008*	0.030	0.75	0.11	0.24	0.12	0.18
IL-17E pla	0.003	0.97	0.16	0.09	0.14	0.12	0.08	0.38
IL-17E cord	0.08		*0.38*	<*0.0001*	*0.19*	*0.04*	0.11	0.26
IL-6 per	−0.05	0.59	0.02	0.78	−0.14	0.14	−0.04	0.64
IL-6 pla	0.03	0.74	−0.02	0.83	0.08	0.40	0.09	0.33
IL-6 cord	−0.01	0.88	0.03	0.70	−0.001	0.98	−0.05	0.55

Significant p values are in italics (p ≤ 0.05)

The Sperman’s rank Order correlation test was used. *Per* Peripheral plasma; *Pla* Placental plasma; *Cord* Cord plasma; *Hb* Haemoglobin level; *IL* Interleukin; *r*_*s*_ Coefficient of correlation

### Regression analysis models on cytokine production

Based on the observed co-variation of parity and mother’s age influencing cytokine levels in the univariate analyses described above, and the potential effects of malaria infection, Hb levels and baby birth weight, the impact of these variables were jointly tested using regression analysis. Results confirmed the univariate analysis. In fact, an association between the decrease in IL-28A, IL-27 levels in peripheral and placental plasma, IL-28A and IL-17E levels in cord plasma and the presence of malaria parasites or pigments in placenta tissue was observed (0.0001 ≤ P ≤ 0.02). The model also showed an association between high levels of Hb and levels of IL-28A, IL-17E and IL-27 in peripheral blood (0.002 ≤ P ≤ 0.018) on one hand, and between high levels of these cytokines in placental plasma, cord plasma and increasing birth weight (0.0001 ≤ P ≤ 0.0027) on the other. With regards to IL-6, regression analysis showed that its peripheral levels increased with decreasing mother’s age (P = 0.05), while high levels of IL-6 in placental plasma associated with an increased parasitaemia between the impression smears (P = 0.03) and the placenta blood (P = 0.045).

## Discussion

Disturbance of cytokines equilibrium has been linked to many pathological disorders, including poor pregnancy outcomes. Although the accumulation of *P. falciparum*-infected erythrocytes in placenta has been shown to cause the alteration of T cells cytokines expression profile in feto-maternal interface [8, 10], the impact of this infection on the plasma levels of some maternal and neonate cytokines known to regulate T cells differentiation and function and how this could affect birth weight remain undefined. This study aimed to investigate the relationship between placental *P. falciparum* infection and the plasma levels of IL-28, IL-27, IL-17E, and IL-6 in Cameroonian maternal and neonate plasma in relation with pregnancy outcomes (birth weight and Hb levels). In general, levels of IL-28A, IL-27 and IL-17E cytokines were observed to be significantly higher in placental and cord plasma than in peripheral plasma. This was as opposed to IL-6 where its levels were higher in peripheral plasma, suggesting that the feto-maternal unit could be the primary source of IL-17E, IL-27 and IL-28A production. Indeed, the syncytiotrophoblast of the placental tissues has been shown to contribute to the production of a number of cytokines, which help in placental growth and fetal tolerance [30, 31]. In addition, cytokines environment in maternal placenta and in fetal cord blood has been shown to have significant effects on baby birth weight [32]. While a previous study demonstrated an association between cord blood cytokine levels of IFN-γ, IL-1β, TNF-α, IL-12p70, IL-10 cytokines, and reduced birth weight [33], this study, for the first time, shows that levels of IL-28A, IL-27 and IL-17E are elevated in cord plasma rather than in maternal peripheral plasma, and correlate positively with increasing baby birth weight. This positive association between birth weight and both the Th1-promoting (IL-27, IL-28A) and Th2-promoting cytokines (IL-17E) is consistent with the findings of Chêne and colleagues, who reported an association between low birth weight and decreasing level of both IFN-γ and IL-5 in intervellous plasma samples from Beninese women [34]. Findings from this study indicate that IL-28A, IL-27 and IL-17E cytokines might be implicated in fetal growth and suggest that a better understanding of the fetal environment mediated by multiple cytokines may be important for optimizing fetal growth outcomes in many settings.

With regard to PM infection, plasma levels of peripheral IL-28A, IL-27 and IL-17E were significantly lower in malaria infected than in malaria non-infected women. All these cytokines correlated negatively and significantly with the parasite densities from impression smears and peripheral blood. Although not significant, negative correlations were also found between placenta blood parasitaemia and plasma levels of placental IL-28A, IL-27 and IL-17E. Similarly, a negative correlation was found between different parasitaemias and cord IL-17E, IL-28A. The presence of both malaria parasites and pigment in the placental tissue associated significantly with low levels of IL-28A in peripheral, placental and cord plasma, as well as the levels of IL-28A in the placental and cord plasma and for a lesser extend IL-17E in cord plasma. These results suggest that PM is associated with decrease maternal IL-28A, IL-27 and IL-17E and neonates’ IL-28A and IL-17E. These results are in agreement with that of a previous study showing an association between pregnancy-associated malaria and reduced plasmacytoid dendritic cells [35], shown to be the main source of IL-28A production [36]. Previous studies have established an association between high malaria parasitaemia and low levels of IFN-γ and IL-17A during pregnancy and at delivery [10, 27]. This is the first study showing the lowering effect of placental *P. falciparum* infection on the IL-28A, IL-27 and IL-17E production. This might be due to the protective effects of the cytokines against the infection. However, although the mechanism is unknown, the association of PM with low levels of cytokines in non-infected cord blood might suggest that PM lowers the levels of these cytokines. Nevertheless, the role of IL-28A in the control of other diseases has been proven. In fact, IL-28A is considered a new approach for the treatment of asthma because its production by the plasmacytoid cells polarizes the immune response through activated Th1 type and the production of the INF-γ [37]. This cytokine, as well as IL-27 and IL-17E, are known to be the main regulators of T cell differentiation and function [26, 38]. Alteration of their expression might be implicated in the decrease of these T cells cytokines (IFN-γ and IL-17A) observed previously. Akin to IL-28A, IL-27 and IL-17 E cytokines, IL-6 also plays a crucial role in T cells differentiation and immune response polarization. It has been shown to inhibit TGF-β-induced IL-10-producing regulatory T cell (Treg) differentiation [17], but together with transforming growth factor (TGF)-β, preferentially promotes the differentiation of IL-17-producing T helper cells (Th17) [18, 39]. Regression analysis showed that its peripheral levels increased with decreasing mother age while high levels of placental plasma IL-6 associated with increased parasitaemias from the impression smears and placenta blood. However, the univariate analysis showed positive correlations between peripheral IL-6 levels and placental blood parasiteamia. A similar trend was observed with peripheral and impression smear parasitaemias, but was not significant. A positive association between malaria infection and plasma levels of this cytokine in women during pregnancy in north-eastern Tanzania was reported [40]. Secretion of IL-6 has been shown to increase progressively in pregnant women from labour until parturition. The non-significance observed in this study could be explained by the fact that the production of this cytokine is influenced by an immunophysiological condition occurring during labour. There was no significant association between plasma levels of IL-6 in placental and neonate blood, as well as placental *P. falciparum* infection.

PM is known to cause maternal anaemia and low birth weight. Thus, the positive association observed between the levels of IL-17E, IL-28A, IL-27, and maternal Hb or between baby birth weight and these cytokines in placental and cord blood, suggest that these cytokines could play protective roles in PM. Indeed, previous studies reported that placental accumulation of IEs might stimulate Th1 cytokine release through macrophages [8, 41] to aid parasite clearance, possibly through complement activation [42], increased phagocytic activity and by the production of reactive oxygen species and nitric oxide (NO) metabolites [43]. Other studies demonstrated that IL-28A and IL-27 cytokines induce the Th1 polarizing cytokines (IFN-γ and IL-12) that protect against malaria in human and animal models [44, 45]. Akin to this, the positive correlation observed between parity and levels of IL-27 and IL-28A in placental plasma support the potential protective role of the three cytokines in PM, as this disease has been shown to be less prevalent and less complicated in multiparous than in primiparous women.

## Conclusions

PM is associated with the downregulation of plasma levels of maternal IL-17E, IL-27, IL-28A, and neonates’ IL-17E and IL-28A cytokines, which might contribute to parasite clearance and enhancement of baby birth weight. These data would contribute to a better understanding of the alteration in cytokine homeostasis in PM patients and their neonates, and provide leads that should help identify potential biomarkers for improved birth weight and therapeutic interventions.

## Abbreviations

PM: placental malaria
IE: infected erythrocyte
IL: interleukin
Th: T helper
NKT: natural killer T cell
IFN-γ: interferon-γ
TGF-β: transforming growth factor β
APC: antigen presenting cell
MFI: mean fluorescence index
RDT: rapid diagnosis test
NO: nitric oxide

## References

[1] Fried M, Duffy PE (1996). Adherence of *Plasmodium falciparum* to chondroitin sulphate A in the human placenta. Science.

[2] Beeson JG, Rogerson SJ, Cooke BM, Reeder JC (2000). Adhesion of *Plasmodium falciparum*-infected erythrocytes to hyaluronic acid in placental malaria. Nat Med.

[3] Davison BB, Cogswell FB, Baskin GB, Falkenstein K, Henson KP, Krogstad D (2000). Placental changes associated with fetal outcome in the *Plasmodium coatneyi/rhesus* monkey model of malaria in pregnancy. Am J Trop Med Hyg.

[4] Ordi J, Menendez C, Ismail MR, Ventura PJ, Palacin A, Kahigwa E (2001). Placental malaria is associated with cell-mediated inflammatory responses with selective absence of natural killer cells. J Infect Dis.

[5] Fried M, Muga RO, Misore AO, Duffy PE (1998). Malaria elicits type 1 cytokines in the human placenta: IFN-gama TNF-α associated with pregnancy outcomes. J Immunol..

[6] Kabyemela ER, Muehlenbachs A, Fried M, Kurtis JD, Mutabingwa TK, Duffy PE (2008). Maternal peripheral blood level of IL-10 as a marker for inflammatory placental malaria. Malar J..

[7] Agudelo OM, Aristizabal BH, Yanow SK, Arango E, Carmona-Fonseca J, Maestre A (2014). Submicroscopic infection of placenta by *Plasmodium* produces Th1/Th2 cytokine imbalance, inflammation and hypoxia in women from north–west Colombia. Malar J..

[8] Suguitan AL, Leke RG, Fouda G, Zhou A, Thuita L, Metenou S, Fogako J, Megnekou R, Taylor DW (2003). Changes in the levels of chemokines and cytokines in the placentas of women with *Plasmodium falciparum* malaria. J Infect Dis.

[9] Raghupathy R (1997). Th1-type immunity is incompatible with successful pregnancy. Immunol Today.

[10] Megnekou R, Djontu JC, Bigoga JD, Lissom A, Magagoum SH (2015). Role of some biomarkers in malaria in women living in Yaoundé, Cameroun. Acta Trop.

[11] Megnekou R, Djontu JC, Bigoga JD, Medou FM, Tenou S, Lissom A (2015). Impact of placental *Plasmodium falciparum* malaria on the profile of some oxidative stress biomarkers in women living in Yaoundé, Cameroon. PLoS ONE.

[12] Lucas S, Ghilardi N, Li J, de Sauvage FJ (2003). IL-27 regulates IL-12 responsiveness of naive 27CD4+ T cells through Stat1-dependant and independent mechanisms. Proc Natl Acad Sci USA.

[13] Takeda A, Hamano S, Yamanaka Hanada AT, Ishibashi T, Mak TW (2003). Cutting edge: role of IL-27/WSX-1 signaling for induction of T-bet through activation of STAT1 during initial Th1commitment. J Immunol..

[14] Kamiya S, Owaki T, Morishima N, Fukai F, Mizuguchi J, Yoshimoto T (2004). An indispensable role for STAT1 in IL-27-induced T-bet expression but not proliferation of naive CD4+ T cells. J Immunol..

[15] Stumhofer JS, Wilson EH, Huang E, Tato CM, Johnson LM, Villarino AV (2006). Interleukin 27 negatively regulates the development of interleukin 17-producing T helper cells during chronic inflammation of the central nervous system. Nat Immunol.

[16] Tomohiro Y, Takayuki Y, Koubun Y, Junichiro M, Kenji N (2007). IL-27 Suppresses Th2 cell development and Th2 cytokines production from polarized Th2 cells: a novel therapeutic way for Th2-mediated allergic inflammation. J Immunol..

[17] Chen W, Jin W, Hardegen N, Lei KJ, Li L, Marinos N (2003). Conversion of peripheral CD4 + CD25- naive T cells to CD4+ CD25+ regulatory T cells by TGF-beta induction of transcription factor Foxp3. J Exp Med.

[18] Bettelli E, Carrier Y, Gao W, Korn T, Strom TB, Oukka M (2006). Reciprocal developmental pathways for the generation of pathogenic effector TH17 and regulatory T cells. Nature.

[19] Dolgachev V, Petersen BC, Budelsky AL, Berlin AA, Lukacs NW (2009). Pulmonary IL-17E (IL-25) production and IL-17RB + myeloid cell-derived Th2 cytokine production are dependent upon stem cell factor-induced responses during chronic allergic pulmonary disease. J Immunol..

[20] Terrier B, Bièche I, Maisonobe T, Laurendeau I, Rosenzwajg M, Kahn JE, Diemert MC (2010). IL-25: a cytokine linking eosinophils and adaptative immunity in Churg-Strauss syndrome. Blood.

[21] Wong CK, Li PW, Lam CW (2007). Intracellular JNK, p38 MAPK and NF-kB regulate IL-25 induced release of cytokines and chemokines from costimulated T helper lymphocytes. Immunol Lett.

[22] Kotenko SV, Gallagher G, Baurin VV, Lewis-Antes A, Shen M, Shah NK (2003). IFN-lambdas mediate antiviral protection through a distinct class II cytokine receptor complex. Nat Immunol.

[23] Meager A, Visvalingam K, Dilger P, Bryan D, Wadhwa M (2005). Biological activity of interleukins-28 and -29: comparison with type I interferons. Cytokine.

[24] Lauterbach H, Bathke B, Gilles Traidl-Hoffmann C, Luber CA, Fejer G (2010). Mouse CD8α^+^ DCs and human BDCA3^+^ DCs are major producers of IFN-λ in response to poly IC. J Exp Med.

[25] Koltsida O, Hausding M, Stavropoulos A, Koch S, Tzelepis G, Ubel C (2011). IL-28A (IFN-λ2) modulates lung DC function to promote Th1 immune skewing and suppress allergic airway disease. EMBO Mol Med..

[26] Hunter CA (2005). New IL-12-family members: IL-23 and IL-27, cytokines with divergent functions. Nat Rev Immunol.

[27] Megnekou R, Lissom A, Bigoga JD, Djontu JC (2015). Effects of pregnancy-associated malaria on T cell cytokines in Cameroonian Women. Scandin J Immunol..

[28] Kimura D, Mivakoda M, Kimura K, Honma K, Hara H, Yoshida H (2016). Interleukin -27—Producing CD4 (+) T cells regulate protective immunity during malaria parasite infection. Immunity.

[29] Rogerson JS, Hviid L, Duffy EP, Leke FGR, Taylor DW (2007). Malaria in pregnancy: pathogenesis and immunity. Lancet.

[30] Bennett WA, Lagoo-Deenadayalan S, Whitworth NS, Stopple JA, Barber WH, Hale E (1999). First trimester human chorionic villi express both immunoregulatory and inflammatory cytokines: a role for interleukin-10 in regulating the cytokine network of pregnancy. Am J Reprod Immunol.

[31] Lin H, Mosmann TR, Guilbert L, Tuntipopipat S, Wegmann TG (1993). Synthesis of T helper2-type cytokines at the maternal foetal interface. J Immunol..

[32] Kabyemela ER, Fried M, Kurtis JD, Mutabingwa KT, Duffy PE (2008). Fetal responses during placental malaria modify the risk of low birth weight. Infect Immun.

[33] Wilkinson A, Pedersen S, Urassa M, Michael D, Andreasen A, Todd J (2015). Elevated umbilical cord cytokines are related to birth size in HIV-exposed and unexposed Infants. FASEB J..

[34] Chêne A, Briand V, Ibitoukou S, Dechayvanne S, Massougbodii A, Deloron P (2014). Placental cytokine and chemokine profiles reflect pregnancy outcomes in women exposed to *Plasmodium falciparum* infection. Infect Immun.

[35] Ibitokou S, Oesterholt M, Brutus L, Borgelle S, Agbowai C, Ezinmègnon S (2012). Peripheral blood cell signatures of *Plasmodium falciparum* infection during pregnancy. PLoS ONE.

[36] Yin Z, Dai J, Deng J, Sheikh F, Natalia M, Shih T (2012). Type III IFNs are produced by and stimulate human plasmacytoid dendritic cells1. J Immunol.

[37] Edwards MR, Johnston SL (2011). Interferon-lambda as a new approach for treatment of allergic asthma?. EMBO Mol Med..

[38] Kastelein RA, Hunter CA, Cua DJ (2007). Discovery and biology of IL-23 and IL-27: related but functionally distinct regulators of inflammation. Annu Rev Immunol.

[39] Veldhoen M, Hocking RJ, Atkins CJ, Locksley RM, Stockinger B (2006). TGFbeta in the context of an inflammatory cytokine milieu supports de novo differentiation of IL-17-producing T cells. Immunity.

[40] Boström S, Ibitokou S, Oesterholt M, Schmiegelow C, Persson JO, Minja D (2012). Biomarkers of *Plasmodium falciparum* infection during pregnancy in women living in north-eastern Tanzania. PLoS ONE.

[41] Fievet N, Moussa M, Tami G, Maubert B, Cot M, Deloron P (2001). *Plasmodium falciparum* induces a Th1/Th2 disequilibrium, favouring the Th1-type pathway, in the human placenta. J Infect Dis.

[42] Mc Donald CR, Tan V, Kain KC (2015). Complement activation in placental malaria. Front Microbial..

[43] Taylor-Robinson AW, Smith EC (1999). A dichotomous role for nitric oxide in protection against blood stage malaria infection. Immunol Let..

[44] Rogerson SJ, Pollina E, Getachew A, Tadesse E, Lema VM, Molyneux ME (2003). Placental monocyte infiltrates in response to *Plasmodium falciparum* malaria infection and their association with adverse pregnancy outcomes. Am J Trop Med Hyg.

[45] Megnekou R, Staalsoe T, Hviid L (2013). Cytokine response to pregnancy-associated recrudescence of *Plasmodium berghei* infection in mice with pre-existing immunity to malaria. Malar J..

